# Successful Management of a Post-Choledochal Cyst Excision Pancreatic Fistula in an Adult Patient: A Case Report and Literature Review on Risk Factors

**DOI:** 10.1055/s-0041-1742175

**Published:** 2022-02-01

**Authors:** Aravinth Anbarasu, Aparna Deshpande

**Affiliations:** 1 Department of General Surgery, Seth GS Medical College & KEM Hospital, Mumbai, Maharashtra, India

**Keywords:** postoperative pancreatic fistula, choledochal cyst

## Abstract

**Introduction** Choledochal cyst is a premalignant condition and surgical excision with biliary enteric anastomosis is the standard of care. Surgical treatment in adults may be difficult due to associated biliary pathology and high incidence of postoperative complications is reported. Postoperative pancreatic fistula (POPF) is a rare early complication following choledochal cyst excision.

**Material and Methods** A 23-year-old male patient was operated for a Todani type IV-A choledochal cyst with anomalous pancreaticobiliary junction. Cyst excision with hepaticojejunostomy was performed. Distal stump closure was technically challenging due to extreme thickening of the cyst wall with neovascularization. On postoperative day 2, patient developed tachycardia and progressive tachypnea with 200 mL of pancreatic fluid in the drain. Endoscopic pancreatic stenting was attempted but was technically not possible. At reexploration, leak from oversewn distal cyst stump was identified and the suture line was reinforced. After the second surgery the patient was hemodynamically stable but continued to have a low output pancreatic fistula for few days which was managed conservatively successfully. We conducted a review of English literature with an aim to identify the risk factors and predictors of pancreatic fistula following cyst excision. An electronic search was performed in Medline and Google Scholar during September 2020 and available literature since January 2000 were reviewed. The keywords used were “pancreatic fistula” and “choledochal cyst.”

**Results** Preoperative cholangiography (magnetic resonance cholangiopancreotography/endoscopic retrograde cholangiopancreatography) is essential to know the extent of cyst and delineate biliary pancreatic junction. Literature review including our case revealed that Todani type I-c, type IV, and forme fruste type of choledochal cyst are at high risk of pancreatic injury and POPF. Recurrent cholangitis makes excision technically more challenging and complete removal is not always possible.

**Conclusion** Postoperative pancreatic fistula can be anticipated in select group of patients with high-risk preoperative findings. Chronic inflammation due to recurrent cholangitis promotes scarring and neovascularization which adds to surgical complexity. Operative technique in these high-risk patients needs further refinement.

Choledochal cyst is a congenital dilatation of the biliary tract which is diagnosed in adulthood in 20% of cases. 1 Adult choledochal cysts are frequently misdiagnosed as biliary stricture, choledocholithiasis, biliary carcinoma, or hepatic abscess. Other complications like pancreatitis, recurrent cholangitis, and concomitant biliary pathology are also more frequent as age advances. It is a premalignant condition with overall risk of cancer reported to be 10 to 15%, and which increases with age. 2 Hence, cyst excision with hepaticoenterostomy (CEHE) is the recommended treatment as soon as the condition is diagnosed. Surgical treatment in adults is much more difficult compared with children due to associated hepatobiliary pathology and incidence of postoperative complication is quite high. 3 Postoperative pancreatic fistula (POPF) is a rare early complication following choledochal cyst excision. Most of these have been managed conservatively with significant associated morbidity. 4 5 6 7 8 Herein, we report our experience in managing POPF in a Todani type IV-A choledochal cyst in a 23-year-old male with operative intervention and a successful outcome. We have also reviewed available literature regarding this unique postoperative complication. An electronic search was performed in Medline and Google Scholar during September 2020 and available literature since January 2000 were reviewed. The keywords used were “pancreatic fistula” and “choledochal cyst.”

## Case Report and Details of Literature Review

**Fig. 1:**
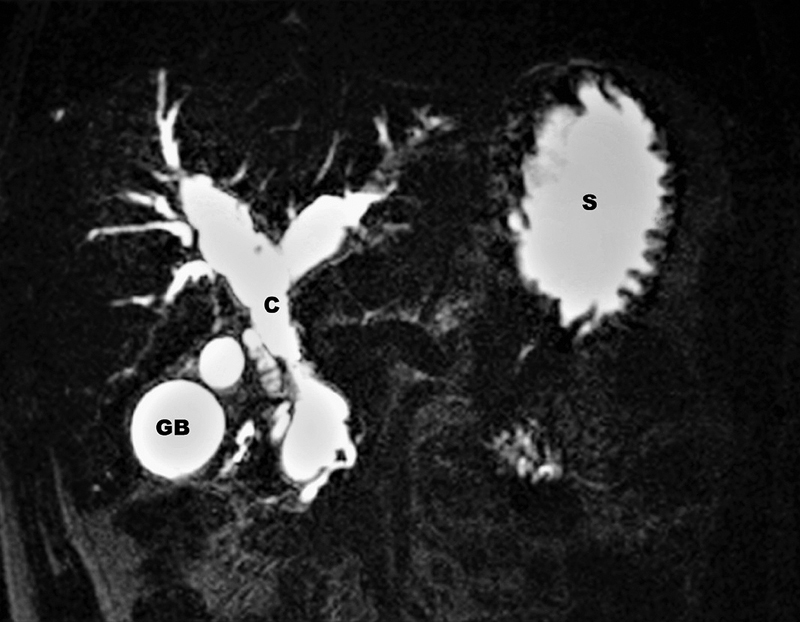
Magnetic resonance cholangiopancreotography (MRCP) showing type IV-A choledochal cyst.

A 23-year-old male presented to us with a history of recurrent right hypochondriac pain, fever, and jaundice, over a period of 12 months and diagnosed as a case of biliary stricture. He had three such episodes each requiring inpatient treatment and intravenous antibiotics. He had undergone endoscopic retrograde cholangiopancreatography (ERCP) and biliary stenting during the first admission and biliary stent was exchanged during subsequent episodes of cholangitis. Patient was referred to our center following the third episode of cholangitis for further management. He was asymptomatic and anicteric at the time of presentation to us. His blood counts and renal and liver function tests were within normal limits except for elevated alkaline phosphatase at 720 IU/L. Ultrasonography revealed a dilated common bile duct with stent in situ. A magnetic resonance cholangiopancreotography (MRCP) revealed a type IV-A choledochal cyst with anomalous pancreaticobiliary duct junction (APBDJ) ( Fig. 1 ). Patient had bilobar intrahepatic disease without any evidence of cirrhosis and malignancy. APBDJ was Komi type IIB where the pancreatic duct joins the cyst at acute angle with dilated common channel.

At laparotomy, a thick-walled choledochal cyst with significant pericholedochal inflammation was encountered leading to technically difficult dissection. In view of this, the proximal dissection was completed first, by isolating the common hepatic duct just below the confluence and disconnecting it. Traction was given to the cyst wall and as much of the cyst wall as possible was excised without damaging the structures in the hepatoduodenal ligament. Part of the posterior wall had to be left behind following Lilly's technique. 9 The cyst wall was extremely thick akin to a coconut shell and dissection external to epicholedochal plexus plane was not possible toward the distal end. This made it difficult to separate the distal part of the cyst from the pancreas. Hence, a small intrapancreatic portion of the cyst of approximately 1cm was left in situ, mucosa of the remnant cyst was cauterized using monopolar cautery. The thickened hard cyst wall stump was oversewn in interrupted fashion using 3–0 delayed absorbable polydioxanone sutures. A Roux-en-Y hepaticojejunostomy restored biliary drainage.

**Fig. 2:**
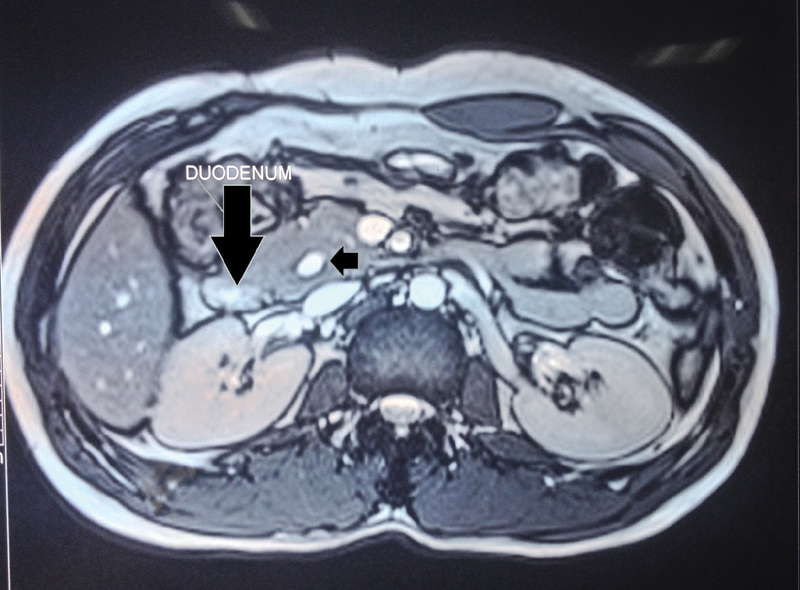
Follow-up magnetic resonance cholangiopancreotography (MRCP): axial image showing small remnant cyst (black arrow).

**Fig. 3:**
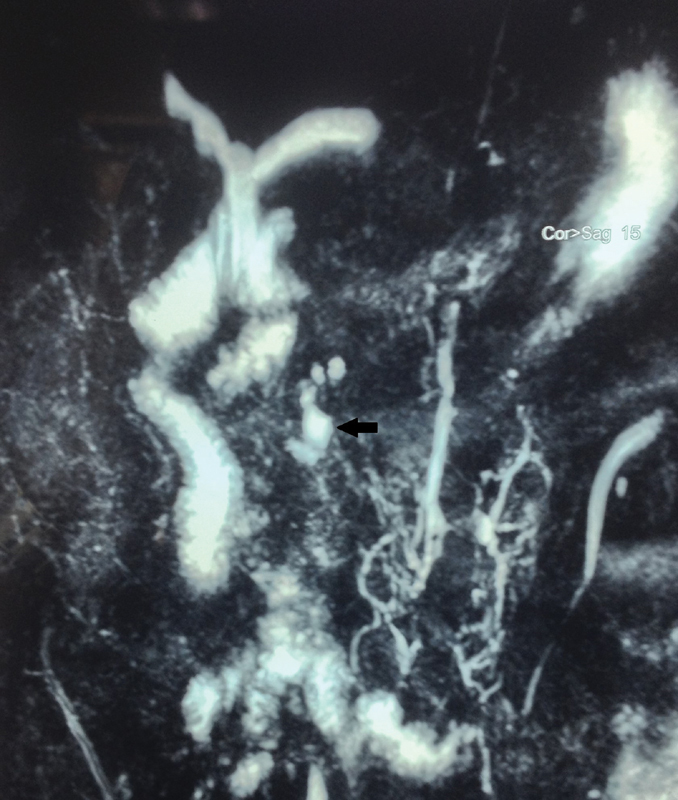
Follow-up magnetic resonance cholangiopancreotography (MRCP): coronal image showing small remnant cyst (black arrow).

Initial postoperative period was uneventful. The drain kept in the Morrison's pouch showed 200 mL of clear fluid on the second postoperative day (POD) which was confirmed biochemically to be a pancreatic fistula. Drain fluid amylase was 12,200 U/L, while serum amylase was normal. Over the next 2 days, the drain output remained at 200 mL/day and patient developed significant tachycardia with a pulse rate of 140/min and right-sided abdominal pain. A contrast-enhanced computed tomography abdomen was performed to look for any changes suggestive of pancreatitis or any localized collections which could be drained by image-guided catheters. However, there were no such findings. An ERCP with pancreatic duct stenting was planned on POD 4 in an attempt to contain the pancreatic leak. It documented a free pancreatic leak around 2 cm from the papilla. However, stenting of pancreatic duct proved technically difficult and was abandoned. Patient was reexplored in view of his persistent tachycardia, tachypnea, and localized abdominal pain, not responding hydration, or other conservative measures. Intraoperatively, there was pancreatic leak from the oversewn choledochal cyst stump. Cyst stump closure was reinforced with deeper polypropylene sutures and fibrin tissue sealant. Patient tolerated the procedure well. Postoperatively, his tachycardia, tachypnea, and pain subsided. From POD 4 after the second surgery the drain output again gradually increased to approximately 100 mL by POD 7 with drain amylase levels of over 5,070 U/L. Patient remained hemodynamically stable and was put on enteral nutrition through nasojejunal tube. Over the next 7 days drain output reduced gradually to less than 10 mL, drain was withdrawn, and subsequently removed on POD 17. Patient was discharged on POD 20 on full oral diet. Follow-up MRCP after 6 months showed a small subcentimetric cyst remnant in pancreas without any pancreatic ductal changes ( Figs. 2 and 3 ).

Literature review in Medline and Google Scholar performed using keywords “pancreatic fistula” AND “Choledochal Cyst” yielded 29 articles. All abstracts were analyzed and relevant articles where full text was available were extracted. Four case series and a case report were reviewed regarding the risk factors and management of POPF. Table 1 summarizes the cases of POPF following choledochal cyst excision as found in literature.

**Table 1:** Literature review and summary of cases with POPF following CEHE

First author	Study type and duration	Number of cases	Cases with POPF	Risk factor identified	Management
Li et al 5 (2001)	Retrospective series in children, 10 years	173	1	Infants < 1 year have higher morbidity	Conservative management
Honda et al 4 (2014)	Retrospective series in children, 7 years	19	6	NA	Not available
Gadelhak et al 6 (2014)	Retrospective series in adults, 20 years	50	1	NA	Conservative management
Liu et al 8 (2017)	Retrospective series in adults, 5 years	54	5	Type 2 distal end (without stenotic distal stump)	CEHE with excision of ampulla of Vater with pancreatic duct plasty
Choi et al 7 (2020)	Case report, adult	1	1	Iatrogenic pancreatic duct injury	Percutaneous drain insertion and endoscopic transmural duodenocystostomy – fulminant protracted recovery
Our case	Case Report, adult	1	1	APBDJ with type 2 distal end, chronic inflammation causing scarring and neovascularization	Reexploration and oversewing of distal cyst stump and reinforcement with fibrin sealant – short recovery period

Abbreviations: APBDJ, anomalous pancreaticobiliary duct junction; CEHE, cyst excision with hepaticoenterostomy; POPF, postoperative pancreatic fistula.

## Discussion

Choledochal cysts are congenital dilatation of biliary tract. They are more common in Asian population and show a female predilection. Adult choledochal cysts when symptomatic, usually present with symptoms mimicking calculus biliary tract disease like recurrent epigastric or right hypochondriac pain, fever, and intermittent jaundice and hence are commonly misdiagnosed as in our case. APBDJ is one of the proposed etiological mechanisms.

MRCP is the preferred noninvasive modality for accurate diagnosis of choledochal cyst. 6 Todani modification of Alonso-Lej classification classifies choledochal cysts into five types based upon the cholangiography features of extent and shape of cysts. Type I followed by IV are the common types of choledochal cysts. 6 Lilly and colleagues described a forme fruste type of choledochal cysts, in which there is abnormal APBDJ without bile duct dilatation and presents with abdominal pain and jaundice. 2 Surgical management depends upon the type of the cyst. CEHE is the treatment for common types of cyst like type I and IV. Complete removal of all cysts is advocated including the distal intrapancreatic portion as the distal remnant may develop malignancy in 0.7 to 6% of cases.

Choledochal cysts without distal stenotic stump like type I-c, certain type IV, and forme fruste cysts are at increased risk of pancreatic injury. Liu et al proposed a distal classification system based on the morphology of the relation of pancreatic duct to distal end of the cyst and the common channel and advocated excision of ampulla of Vater with pancreatic duct plasty along with CEHE for cysts without distal stenotic stump. 8

Distal dissection of intrapancreatic portion of the cyst is technically tedious in the setting of recurrent cholangitis due to scarring and neovascularization. At the distal end, dissection external to epicholedochal plexus is recommended to avoid pancreatic injury. 10 11 But this technique was not possible in our case. Partial cyst excision leaving behind the intrapancreatic portion as advised by Diao et al in select group of patients with distal stenotic stump, can help avoiding pancreatic injury with less likelihood of POPF. 12 In our patient, we had to resort to this option, in spite of not having distal stenotic stump due to the technical difficulty in distal stump dissection in the setting of chronic inflammation. Some authors have described selective use of intraoperative cyst endoscopy or maneuver like inserting a probe into pancreatic duct through a duodenotomy in difficult cases to avoid pancreatic duct injury. 3 13 Early postoperative complication rate following CEHE is around 9.3%, the most common complication being biliary leakage (5.7%). 5 Comparatively, pancreatic fistula is a rare complication in the postoperative period following choledochal cyst excision. Reported incidence of pancreatic injury following choledochal cyst excision is 2 to 6%. 6 Pancreatic fistula usually presents as characteristic turbid white-colored drainage from the intraoperatively placed drain in the first postoperative week. Most reported fistulas are managed conservatively and require no additional intervention. Uncontrolled fistulas have been managed by endoscopic pancreatic duct stenting or image-guided catheters. Intractable pancreatic fistula can induce catastrophic complications, resulting in prolonged hospital stay and reinterventions. 7 In our case, operative intervention was needed for following reasons: (1) ERCP was unsuccessful, (2) there was no localized collection to allow image-guided drainage as the symptoms came very early, and (3) the patient's parameters precluded continuing conservative management.

Analyzing the 14 cases of POPF following CEHE from our review, APBDJ with type 2 distal end and iatrogenic pancreatic duct injury during distal dissection were the specific risk factors for POPF in adults.

In conclusion, POPF after cholecodchal cyst excision is a rare but severe complication. Difficult dissection and pancreatic injury should be anticipated in cysts presenting with recurrent cholangitis and in cysts without distal stenotic stump (type IC, forme fruste, and certain type IV cysts). APBDJ with a nonstenotic distal end and difficulty in approximation of the thickened distal stump all could have played a role in development of POPF in our patient. Operative techniques in these cysts with high-risk features need further evaluation.
